# SNP-SNP interactions of three new pri-miRNAs with the target gene *PGC* and multidimensional analysis of *H. pylori* in the gastric cancer/atrophic gastritis risk in a Chinese population

**DOI:** 10.18632/oncotarget.8057

**Published:** 2016-03-14

**Authors:** Qian Xu, Ye-feng Wu, Ying Li, Cai-yun He, Li-ping Sun, Jing-wei Liu, Yuan Yuan

**Affiliations:** ^1^ Tumor Etiology and Screening Department of Cancer Institute and General Surgery, The First Affiliated Hospital of China Medical University, and Key Laboratory of Cancer Etiology and Prevention (China Medical University), Liaoning Provincial Education Department, Shenyang 110001, China

**Keywords:** miRNA, pepsinogen C, polymorphisms, H. pylori, gastric cancer

## Abstract

Gastric cancer (GC) is a multistep complex disease involving multiple genes, and gene–gene interactions have a greater effect than a single gene in determining cancer susceptibility. This study aimed to explore the interaction of the let-7e rs8111742, miR-365b rs121224, and miR-4795 rs1002765 single nucleotide polymorphisms (SNPs) with SNPs of the predicted target gene *PGC* and *Helicobacter pylori* status in GC and atrophic gastritis (AG) risk. Three miRNA SNPs and seven *PGC* SNPs were detected in 2448 cases using the Sequenom MassArray platform. Two pairwise combinations of miRNA and *PGC* SNPs were associated with increased AG risk (let-7e rs8111742 – *PGC* rs6458238 and miR-4795 rs1002765 – *PGC* rs9471643). Singly, miR-365b rs121224 and *PGC* rs6912200 had no effect individually but in combination they demonstrated an epistatic interaction associated with AG risk. Similarly, let-7e rs8111742 and miR-4795 rs1002765 SNPs interacted with *H. pylori* infection to increase GC risk (rs8111742: *P*_interactio*n*_ = 0.024; rs1002765: *P*_interactio*n*_ = 0.031, respectively). A three-dimensional interaction analysis found miR-4795 rs1002765, *PGC* rs9471643, and *H. pylori* infection positively interacted to increase AG risk (*P*_interactio*n* =_ 0.027). Also, let-7e rs8111742, *PGC* rs6458238, and *H. pylori* infection positively interacted to increase GC risk (*P*_interactio*n* =_ 0.036). Furthermore, both of these three-dimensional interactions had a dosage–effect correspondence (*P*_trend_ < 0.001) and were verified by MDR. In conclusion, the miRNAs SNPs (let-7e rs8111742 and miR-4795 rs1002765) might have more superior efficiency when combined with *PGC* SNPs and/or *H. pylori* for GC or AG risk than a single SNP on its own.

## INTRODUCTION

Although single nucleotide polymorphisms (SNPs) are potential predicting biomarkers of cancer risk, they have limited use for multistage complex diseases involving multiple genes [1–3]. Several studies have reported that gene–gene interactions are more important than single genes in promoting cancer susceptibility [3–5]. For example, polymorphisms that individually have a weak effect can have a strong effect when acting together [6]. Similarly, in epistasis, which is a phenomenon that consists of the effect of complex interactions, is greater than the main effects of any single susceptibility gene [5, 7].

MiRNAs bind to the 3′-untranslated regions of coding genes to suppress or downregulate gene expression. Translational regulation is performed by a network of miRNAs and target genes such that one miRNA can regulate multiple different genes, or one target gene can be regulated by several miRNAs [8]. In recent years, several studies have simultaneously screened miRNA SNPs and target gene SNPs, and interactions between specific pairs to promote carcinogenic effect have been identified [9].

We previously screened miRNAs targeting a single gene, and identified three different miRNA polymorphisms (pri-let-7e rs8111742, pri-miR-365b rs121224 and pri-miR-4795 rs1002765) that had no or only weak effects on gastric cancer (GC) and atrophic gastritis (AG) risk (unpublished data). However, these three miRNA SNPs were all located in the promoter region of miRNA genes (rs8111742 at −565 bp of the let-7e gene, rs121224 at −430 bp of the miR-365b gene, and rs1002765 at −1635 bp of the miR-4795 gene), suggesting that they may be functional polymorphisms. All miRNAs were predicted to stimulate/inhibit pepsinogen C (*PGC*) gene, which is expressed by terminally differentiated gastric mucosa cells. *PGC* is a diagnostic biomarker for GC and AG (precancerous disease) [10]. Our colleagues investigated the association between each of the seven *PGC* SNPs and GC and/or AG, and found that five *PGC* SNPs were linked to GC or AG risk, while two (*PGC* rs6912200 and rs6941539) had no significant association with cancer risk [11, 12]. We therefore hypothesized that the former may be optimal candidates for investigating potential SNP–SNP interactions for miRNA SNPs-*PGC* SNPs at two or more genetic loci. It was unclear whether there were interactions among these SNPs and whether detecting combinations of them could enhance the risk prediction for GC or AG.

Gastric carcinogenesis may occur via a network of molecular changes caused by both environmental and hereditary factors. *Helicobacter pylori* is the most important environmental factor; *H. pylori* infection can cause AG, intestinal metaplasia, or GC, and also has a non-negligible interaction with hereditary factors to increase host susceptibility [12]. *H. pylori* infection might promote the progression from normal gastric mucosa to AG, and to GC. Thus, there may be a three-way interaction between specific miRNA and target gene SNPs and *H. pylori* infection. Identifying such an interaction may provide a reliable predicting biomarker for gastric cancer development [14], and may could help to comprehend how miRNA, target gene and *H. pylori* infection together regulate gastric carcinogenesis.

This study was based on previous a case–control study in a Chinese population (unpublished data) and investigated two- and three-dimensional interactions between miRNA SNPs, SNPs in its predicted target gene *PGC*, and *H. pylori* infection status. It may identify combination biomarkers for early GC.

## RESULTS

### Demographic and geographic characteristics

The demographic and geographic characteristics of study participants are shown in Supplementary Table S1 (including age, sex, and *H. pylori* infection status). There was no significant difference in the age and sex distribution among the GC, AG, and control groups.

### Relationship between single SNPs and GC/AG risk

We previously investigated associations of three miRNA polymorphisms with seven *PGC* polymorphisms affecting the risk of GC and AG in a Chinese population (unpublished data). We found no significant association of let-7e rs8111742, miR-365b rs121224, and miR-4795 rs1002765 with GC and/or AG risk (*P* > 0.05). The *PGC* rs4711690 CG+CC genotype, rs6458238 GA+AA genotype, and rs3789210 CG+GG genotype were associated with a decreased AG risk (*P* = 0.008, OR = 0.78; *P* = 0.015, OR = 0.73; and *P* = 0.048, OR = 0.78, respectively). The *PGC* rs6939861 AG+AA genotype was linked to an increased risk of GC and AG (GC risk *P* = 0.015, OR = 1.32; AG risk *P* = 0.014, OR = 1.30). Furthermore, the *PGC* rs9471643 GC genotype was associated with a significantly decreased GC risk compared with the CC+GG genotype (*P* = 0.025, OR = 0.79). However, there was no significant link between the *PGC* rs6912200 and rs6941539 polymorphisms and the risk of both GC and AG (*P* > 0.05) [11, 12].

### Pairwise interactions between miRNA and PGC SNPs

To further investigate the interaction of three miRNA SNPs and SNPs of their predicted target gene *PGC*, we first analyzed pairwise interaction effects using significant effect models in the main effect analysis (Table 1). MiRNA SNPs all used a recessive model, and all *PGC* SNPs except for rs9471643 and rs6912200 used a dominant model. In addition, *PGC* rs9471643 used a complete overdominant model, while *PGC* rs6912200 used a recessive model based on previous data [15]. We found four pairwise combinations that influenced AG risk: one combination negatively interacted with the AG risk, while the other three all positively interacted with the AG risk. The let-7e rs8111742 – *PGC* rs6458238, miR-365b rs121224 – *PGC* rs691220, miR-4795 rs1002765 – *PGC* rs9471643 pairwise combinations positively interacted with the AG risk (*P*_interactio*n*_ = 0.025, interaction index = 4.89; *P*_interactio*n*_ = 0.022, interaction index = 2.02; and *P*_interactio*n*_ = 0.006, interaction index = 2.23, respectively). The let-7e rs8111742 – *PGC* rs6941539 combination had a negative pairwise interaction in AG risk (*P*_interactio*n*_ = 0.034, interaction index = 0.40). However, none of the miRNA SNP – *PGC* SNP combinations had a significant interaction (*P* > 0.05) with the GC risk.

**Table 1:** The interaction of three miRNA polymorphisms with *PGC* polymorphisms in the risk of gastric cancer/atrophic gastritisa^a^

		let-7e rs8111742		miR-365b rs121224		miR-4795 rs1002765	
		GA+GG	AA	CG+CC	GG	GA+GG	AA
**AG vs CON (n=862 Vs. 862)**							
*PGC* rs4711690							
CG+GG	Case/Control	296/357	22/30	249/315	69/72	264/324	54/63
	OR(95%CI)	1(Ref)	0.88 (0.50-1.56)	1(Ref)	1.21 (0.84-1.75)	1(Ref)	0.58 (0.23-1.47)
CC	Case/Control	449/439	35/30	387/377	97/92	408/397	76/72
	OR(95%CI)	1.24 (1.01-1.52)	1.40 (0.84-2.34)	1.31 (1.05-1.62)	1.33 (0.96-1.85)	0.60 (0.25-1.44)	0.60 (0.26-1.40)
		*P*_interaction_=0.699		*P*_interaction_=0.908		*P*_interaction_=0.555	
		interaction index=1.17		interaction index=0.97		interaction index=1.19	
*PGC* rs6458238							
GA+AA	Case/Control	108/135	3/14	94/120	17/29	92/125	19/24
	OR(95%CI)	1(Ref)	0.27(0.07-0.95)	1(Ref)	0.74(0.39-1.43)	1(Ref)	0.94(0.49-1.81)
GG	Case/Control	638/659	54/46	542/571	150/134	579/594	113/111
	OR(95%CI)	1.21(0.92-1.59)	1.46(0.91-2.33)	1.21(0.90-1.62)	1.42(0.99-2.03)	1.31(0.68-2.53)	1.23(0.67-2.28)
		***P*_interaction_=0.025**		*P*_interaction_=0.304		*P*_interaction_=0.751	
		**interaction index=4.89**		interaction index=1.48		interaction index=0.88	
*PGC* rs9471643							
GC	Case/Control	273/312	19/22	217/262	75/72	247/268	45/66
	OR(95%CI)	1(Ref)	0.99(0.52-1.86)	1(Ref)	1.26(0.87-1.82)	1(Ref)	0.74(0.49-1.12)
GG+CC	Case/Control	469/484	38/39	415/433	92/90	420/455	87/68
	OR(95%CI)	1.12(0.91-1.37)	1.11(0.69-1.79)	1.17(0.93-1.46)	1.23(0.88-1.74)	1.01(0.81-1.25)	1.43(0.99-2.06)
		*P*_interaction_=0.773		*P*_interaction_=0.960		***P*_interaction_=0.006**	
		interaction index=0.89		interaction index=1.01		**interaction index=2.23**	
*PGC* rs3789210							
GG+CC	Case/Control	172/201	13/11	144/176	41/36	157/171	28/41
	OR(95%CI)	1(Ref)	1.38(0.60-3.16)	1(Ref)	1.39(0.85-2.29)	1(Ref)	0.74(0.44-1.26)
GC	Case/Control	629/599	48/51	541/522	136/128	563/556	114/94
	OR(95%CI)	1.24(0.98-1.72)	1.10(0.71-1.71)	1.28(0.99-1.64)	1.30(0.94-1.80)	1.11(0.87-1.42)	1.35(0.95-1.92)
		*P*_interaction_=0.392		*P*_interaction_=0.307		*P*_interaction_=0.131	
		interaction index=0.65		interaction index=0.73		interaction index=1.64	
*PGC* rs6912200							
TC+CC	Case/Control	602/592	43/52	514/512	131/132	533/540	112/104
	OR(95%CI)	1(Ref)	0.81(0.53-1.23)	1(Ref)	0.98(0.75-1.29)	1(Ref)	1.11(0.83-1.49)
TT	Case/Control	196/204	18/10	169/184	45/30	185/183	29/31
	OR(95%CI)	0.95(0.75-1.19)	1.76(0.81-3.85)	0.92(0.72-1.17)	1.49(0.92-2.40)	1.03(0.81-1.30)	0.95(0.56-1.59)
		*P*_interaction_=0.205		***P*_interaction_=0.022**		*P*_interaction_=0.907	
		interaction index=1.84		**interaction index=2.02**		interaction index=0.96	
*PGC* rs6939861							
GG	Case/Control	310/354	25/19	264/298	71/75	278/316	57/57
	OR(95%CI)	1(Ref)	1.50(0.81-2.78)	1(Ref)	1.07(0.74-1.54)	1(Ref)	1.14(0.76-1.70)
GA+AA	Case/Control	467/408	34/40	400/369	101/79	422/375	79/73
	OR(95%CI)	1.32(1.08-1.62)	0.97(0.60-1.57)	1.24(1.00-1.54)	1.44(1.03-2.02)	1.29(1.04-1.59)	1.27(0.88-1.81)
		*P*_interaction_=0.050		*P*_interaction_=0.923		*P*_interaction_=0.414	
		interaction index=0.44		interaction index=1.03		interaction index=0.79	
*PGC* rs6941539							
CC	Case/Control	555/586	45/38	474/512	122/112	506/522	90/102
	OR(95%CI)	1(Ref)	1.26(0.80-1.96)	1(Ref)	1.17(0.88-1.56)	1(Ref)	0.92(0.67-1.25)
CT+TT	Case/Control	244/209	15/24	206/182	53/51	210/200	49/33
	OR(95%CI)	1.25(1.00-1.56)	0.66(0.34-1.28)	1.23(0.97-1.56)	1.12(0.75-1.68)	1.09(0.86-1.37)	1.58(0.99-2.50)
		***P*_interaction_=0.034**		*P*_interaction_=0.269		*P*_interaction_=0.121	
		**interaction index=0.40**		interaction index=0.74		interaction index=1.62	
**GC vs CON (n=724 Vs. 729)**							
*PGC* rs4711690							
CG+GG	Case/Control	195/296	19/26	174/262	40/60	181/274	33/48
	OR(95%CI)	1(Ref)	1.11(0.60-2.05)	1(Ref)	1.00(0.64-1.56)	1(Ref)	1.06(0.66-1.72)
CC	Case/Control	280/377	25/27	256/326	49/78	342/264	41/62
	OR(95%CI)	1.13(0.89-1.44)	1.40(0.79-2.49)	1.19(0.92-1.53)	0.94(0.63-1.41)	1.18(0.92-1.51)	1.02(0.66-1.58)
		*P*_interaction_=0.376		*P*_interaction_=0.625		*P*_interaction_=0.563	
		interaction index=1.48		interaction index=0.86		interaction index=0.82	
*PGC* rs6458238							
GA+AA	Case/Control	81/117	9/11	70/103	20/25	75/108	15/20
	OR(95%CI)	1(Ref)	1.17(0.46-2.96)	1(Ref)	1.17(0.60-2.26)	1(Ref)	1.07(0.52-2.22)
GG	Case/Control	392/552	35/42	358/482	69/112	369/504	58/90
	OR(95%CI)	1.02(0.75-1.40)	1.19(0.70-2.03)	1.09(0.78-1.52)	0.90(0.59-1.38)	1.05(0.76-1.45)	0.94(0.60-1.47)
		*P*_interaction_=0.561		*P*_interaction_=0.202		*P*_interaction_=0.802	
		interaction index=0.73		interaction index=0.61		interaction index=0.90	
*PGC* rs9471643							
GC	Case/Control	162/254	13/20	140/210	35/64	152/220	23/54
	OR(95%CI)	1(Ref)	1.02(0.49-2.11)	1(Ref)	0.82(0.52-1.31)	1(Ref)	0.62(0.36-1.05)
GG+CC	Case/Control	311/416	31/34	288/378	54/72	292/395	50/55
	OR(95%CI)	1.18(0.93-1.51)	1.43(0.85-2.42)	1.16(0.89-1.50)	1.13(0.75-1.70)	1.08(0.83-1.39)	1.37(0.88-2.12)
		*P*_interaction_=0.547		*P*_interaction_=0.295		*P*_interaction_=0.062	
		interaction index=1.33		interaction index=1.40		interaction index=1.94	
*PGC* rs3789210							
CG+GG	Case/Control	165/164	17/10	148/149	34/25	159/140	23/34
	OR(95%CI)	1(Ref)	1.69(0.75-3.80)	1(Ref)	1.37(0.78-2.41)	1(Ref)	0.60(0.34-1.06)
CC	Case/Control	501/510	41/45	449/442	93/113	462/479	80/76
	OR(95%CI)	0.98(0.77-1.26)	0.91(0.56-1.46)	1.03(0.78-1.34)	0.83(0.58-1.18)	0.85(0.66-1.11)	0.95(0.65-1.41)
		*P*_interaction_=0.249		*P*_interaction_=0.091		*P*_interaction_=0.099	
		interaction index=0.57		interaction index=0.57		interaction index=1.79	
*PGC* rs6912200							
TC+CC	Case/Control	498/495	41/45	444/433	95/107	459/455	80/85
	OR(95%CI)	1(Ref)	0.90(0.58-1.40)	1(Ref)	0.86(0.63-1.17)	1(Ref)	0.95(0.68-1.33)
TT	Case/Control	164/175	17/10	149/156	32/29	158/160	23/25
	OR(95%CI)	0.93(0.73-1.19)	1.68(0.76-3.70)	0.93(0.72-1.21)	1.07(0.64-1.80)	0.98(0.76-1.27)	0.91(0.51-1.63)
		*P*_interaction_=0.158		*P*_interaction_=0.227		*P*_interaction_=0.814	
		interaction index=1.98		interaction index=1.49		interaction index=1.09	
*PGC* rs6939861							
GG	Case/Control	262/301	18/16	233/251	47/66	516/548	90/97
	OR(95%CI)	1(Ref)	1.29(0.65-2.59)	1(Ref)	0.77(0.51-1.16)	1(Ref)	0.84(0.53-1.35)
GA+AA	Case/Control	364/345	36/36	326/317	74/64	68/43	6/10
	OR(95%CI)	1.23(0.98-1.53)	1.15(0.70-1.88)	1.12(0.89-1.42)	1.25(0.85-1.82)	1.18(0.94-1.49)	1.14(0.77-1.70)
		*P*_interaction_=0.426		*P*_interaction_=0.231		*P*_interaction_=0.940	
		interaction index=0.70		interaction index=1.42		interaction index=1.02	
*PGC* rs6941539							
CC	Case/Control	491/496	44/33	438/432	97/97	465/445	70/84
	OR(95%CI)	1(Ref)	1.34(0.84-2.14)	1(Ref)	0.98(0.72-1.34)	1(Ref)	0.81(0.57-1.14)
CT+TT	Case/Control	174/173	13/22	158/155	29/40	154/169	33/26
	OR(95%CI)	1.02(0.80-1.31)	0.60(0.30-1.19)	1.01(0.78-1.31)	0.71(0.43-1.17)	0.88(0.68-1.13)	1.26(0.74-2.15)
		*P*_interaction_=0.061		*P*_interaction_=0.454		*P*_interaction_=0.189	
		interaction index=0.43		interaction index=0.79		interaction index=1.58	

Note:

a *P* for interaction was used Logistic Regession adjusted by gender, age and *H. pylori* infection status; CON: controls; AG: atrophic gastritis; GC: gastric cancer.

### Epistatic effects of two-way miRNA SNP – PGC SNP interactions

Among the four pairwise interactions involving three miRNA SNPs and 4 *PGC* SNPs, there was no significant association between the three miRNA SNPs and the*PGC* rs6912200 and *PGC* rs6941539 polymorphisms in the main effect analysis [12]. We therefore investigated epistatic effects between the pairs of interacting SNPs (Table 2). Regarding miR-365b rs121224 and *PGC* rs6912200, the rs121224 GG genotype and the rs6912200 TT genotype were both associated with an increased risk of AG (OR = 2.28 and 1.75, respectively), but only if they were both present. Regarding miR-4795 rs1002765, the AA genotype was associated with an increased risk of AG (OR = 1.49), but only in the presence of the *PGC* rs9471643 GG+CC genotype. Regarding *PGC* rs6941539, the CT+TT genotype was associated with a decreased risk of GC (OR = 0.38), but only in the presence of the let-7e rs8111742 AA genotype. Other polymorphisms such as *PGC* rs6458238 were found to be associated with an increased AG risk in our previous study, but showed a stronger interaction with AG risk (OR = 6.95) in the presence of the let-7e rs8111742 AA genotype in the present study. The *PGC* rs9471643 SNP was also associated with a decreased risk of GC risk in our previous study. However, in this study it was found to be linked to a reduced GC risk and reduced AG risk (OR = 0.46 and 0.45, respectively). These observations suggest that miR-365b rs121224, miR-4795 rs1002765, and both *PGC* rs6912200 and rs6941539 SNPs have no effect individually but demonstrate pairwise epistatic interactions.

**Table 2:** Epistatic effect of pair-wise interacting factors on the risks of gastric cancer and atrophic gastritis

Interacted pair-wise SNPs	Comparison	Subset	AG vs. CON		GC vs. CON	
			*P*	OR(95%CI)	*P*	OR(95%CI)
let-7e rs8111742 interacted with *PGC* rs6458238	let7-e rs8111742 AA vs. GA+GG	PGC rs6458238 GA+AA	0.056	0.28(0.08-1.04)	0.457	1.44(0.55-3.75)
		PGC rs6458238 GG	0.248	1.29(0.84-1.99)	0.757	1.08(0.66-1.76)
	PGC rs6458238 GG vs. GA+AA	let-7e rs8111742 GA+GG	0.198	1.21(0.91-1.62)	0.709	1.06(0.77-1.47)
		let-7e rs8111742 AA	**0.008**	**6.95(1.65-29.24)**	0.168	0.43(0.13-1.43)
let-7e rs8111742 interacted with *PGC* rs6941539	let-7e rs8111742 AA vs. GA+GG	PGC rs6941539 CC	0.062	0.52(0.26-1.04)	0.107	0.54(0.26-1.14)
		PGC rs6941539 CT+TT	0.270	1.31(0.81-2.10)	0.267	1.32(0.81-2.14)
	PGC rs6941539 CT+TT vs. CC	let-7e rs8111742 GA+GG	0.061	1.25(0.99-1.57)	0.890	1.02(0.79-1.31)
		let-7e rs8111742 AA	0.089	0.48(0.21-1.12)	**0.042**	**0.38(0.15-0.97)**
miR-4795 rs1002765 interacted with *PGC* rs9471643	miR-4795 rs1002765 AA vs. GA+GG	PGC rs9471643 GG+CC	**0.033**	**1.49(1.03-2.16)**	0.361	1.22(0.79-1.89)
		PGC rs9471643 GC	0.059	0.65(0.42-1.02)	0.124	0.65(0.38-1.12)
	PGC rs9471643 GC vs. GG+CC	miR-4795 rs1002765 GA+GG	0.691	0.95(0.76-1.20)	0.436	0.90(0.69-1.18)
		miR-4795 rs1002765 AA	**0.003**	**0.45(0.27-0.76)**	**0.016**	**0.46(0.25-0.87)**
miR-365b rs121224 interacted with *PGC* rs6912200	miR-365b rs121224 GG vs. CG+CC	PGC rs6912200 TC+CC	0.821	0.97(0.73-1.28)	0.346	0.86(0.63-1.18)
		PGC rs6912200 TT	**0.004**	**2.28(1.29-4.00)**	0.385	1.29(0.73-2.31)
	PGC rs6912200 TT vs. TC+CC	miR-365b rs121224 CG+CC	0.398	0.90(0.69-1.16)	0.672	0.94(0.72-1.24)
		miR-365b rs121224 GG	**0.045**	**1.75(1.01-3.03)**	0.254	1.41(0.78-2.56)

Note: All tests were adjusted by age, sex and *H. pylori* infection. Statistically significant associations were highlighted in bold (*P* values <0.05). Note: GC, gastric cancer; AG, atrophic gastritis; CON, controls.

### Pairwise interactions between individual miRNA SNPs

We also investigated interactions between different miRNA SNPs but found no significant interaction with either the GC or AG risk (*P* > 0.05; Supplementary Table S2).

### Multidimensional analysis of SNP–SNP interactions between miRNA and PGC with H. pylori status

We analyzed the four two-way interaction combinations stratified by *H. pylori* status, and found that in the *H. pylori* (+) subgroup, the let-7e rs8111742 – *PGC* rs6941539 combination showed negative interactions with both GC and AG risk (interaction index = 0.23 and 0.16). Moreover, the miR-365b rs121224 – *PGC* rs691220 and miR-4795 rs1002765 – *PGC* rs9471643 pairwise combinations demonstrated positive interactions with AG risk in the *H. pylori* (−) subgroup (interaction index = 2.16 and 4.10, respectively, Table 3).

**Table 3:** The interaction of miRNA SNP-*PGC* SNP stratified by *H. pylori* infection in the risk of atrophic gastritis^a^

SNP genotypes		AG vs. CON		GC vs. CON	
		*P*	OR(95%CI)	*P*	OR(95%CI)
*H. pylori* (−)					
let-7e rs8111742-*PGC* rs6458238					
GA+GG	GA+AA		1(Ref)		1(Ref)
GA+GG	GG	0.772	1.06(0.72-1.55)	0.857	1.04(0.68-1.59)
AA	GA+AA	0.128	0.20(0.03-1.59)	0.362	1.64(0.57-4.73)
AA	GG	0.518	1.23(0.66-2.30)	0.059	0.42(0.17-1.03)
		*P*_interaction_=0.112		*P*_interaction_=0.049	
		interaction index=5.76		interaction index=0.26	
let-7e rs8111742-*PGC* rs6941539					
GA+GG	CC		1(Ref)		1(Ref)
GA+GG	CT+TT	0.074	1.31(0.97-1.77)	0.800	1.04(0.76-1.44)
AA	CC	0.928	1.03(0.56-1.90)	0.381	0.75(0.39-1.43)
AA	CT+TT	0.668	0.82(0.33-2.02)	0.266	0.58(0.22-1.52)
		*P*_interaction_=0.366		*P*_interaction_=0.593	
		interaction index=0.60		interaction index=0.73	
miR-4795 rs1002765-*PGC* rs9471643					
GA+GG	GC		1(Ref)		1(Ref)
GA+GG	GG+CC	0.656	0.93(0.69-1.27)	0.695	0.93(0.66-1.32)
AA	GC	0.001	0.53(0.27-1.05)	0.476	0.78(0.40-1.54)
AA	GG+CC	0.096	1.99(1.23-3.22)	0.092	1.64(0.92-2.92)
		***P*_interaction_=0.001**		*P*_interaction_=0.075	
		**interaction index=4.10**		interaction index=2.21	
miR-365b rs121224-*PGC* rs6912200					
CG+CC	TC+CC		1(Ref)		1(Ref)
CG+CC	TT	0.023	0.67(0.47-0.95)	0.339	0.85(0.60-1.19)
GG	TC+CC	1.000	1.00(0.69-1.46)	0.962	0.99(0.66-1.48)
GG	TT	0.216	1.45(0.81-2.60)	0.935	1.03(0.55-1.93)
		***P*_interaction_=0.042**		*P*_interaction_=0.604	
		**interaction index=2.16**		interaction index=1.23	
*H. pylori* (+)					
let-7e rs8111742-*PGC* rs6458238					
GA+GG	GA+AA		1(Ref)		1(Ref)
GA+GG	GG	0.109	1.43(0.92-2.20)	0.735	1.09(0.67-1.78)
AA	GA+AA	0.220	0.34(0.06-1.92)	0.862	0.84(0.11-6.24)
AA	GG	0.050	2.32(1.00-5.37)	0.012	3.35(1.31-8.57)
		*P*_interaction_=0.112		*P*_interaction_=0.248	
		interaction index=4.68		interaction index=3.64	
let-7e rs8111742-*PGC* rs6941539					
GA+GG	CC		1(Ref)		1(Ref)
GA+GG	CT+TT	0.393	1.17(0.82-1.67)	0.883	0.97(0.65-1.46)
AA	CC	0.113	1.99(0.85-4.65)	0.010	4.04(1.39-11.74)
AA	CT+TT	0.170	0.50(0.18-1.35)	0.284	0.56(0.19-1.62)
		***P*_interaction_=0.028**		***P*_interaction_=0.018**	
		**interaction index=0.23**		**interaction index=0.16**	
miR-4795 rs1002765-*PGC* rs9471643					
GA+GG	GG+CC		1(Ref)		1(Ref)
GA+GG	GC	0.254	1.23(0.86-1.75)	0.088	1.44(0.95-2.17)
AA	GG+CC	0.442	0.78(0.43-1.41)	0.076	0.46(0.19-1.09)
AA	GC	0.119	0.99(0.56-1.76)	0.912	1.44(0.95-2.17)
		*P*_interaction_=0.981		*P*_interaction_=0.486	
		interaction index=1.01		interaction index=1.48	
miR-365b rs121224-*PGC* rs6912200					
CG+CC	TC+CC		1(Ref)		1(Ref)
CG+CC	TT	0.177	1.32(0.88-1.98)	0.560	1.14(0.73-1.79)
GG	TC+CC	0.690	0.92(0.60-1.40)	0.137	0.69(0.42-1.13)
GG	TT	0.064	2.79(0.94-8.24)	0.278	1.87(0.60-5.81)
		*P*_interaction_=0.171		*P*_interaction_=0.196	
		interaction index=2.32		interaction index=2.33	

Note:

a *P* for interaction was used Logistic Regession adjusted by gender and age. CON, controls; AG, atrophic gastritis; GC, gastric cancer; NA, not available.

The stratified analyses prompted us to further analyze the interaction between miRNA polymorphisms and *H. pylori* positivity. We found that the let-7e rs8111742 and *H. pylori* (+) interaction and the miR-4795 rs1002765 and *H. pylori* (+) interaction had effects on GC risk (*P*_interactio*n*_ = 0.024, interaction index = 2.75; *P*_interactio*n*_ = 0.031, interaction index = 0.51, respectively; Table 4).

**Table 4:** The interaction of three miRNA polymorphisms with *H.pylori* infection status in the risk of gastric cancer/atrophic gastritis^a^

		let-7e rs8111742		miR-365b rs121224		miR-4795 rs1002765	
		GA+GG	AA	CG+CC	GG	GA+GG	AA
**AG vs CON (n=862 Vs. 862)**							
*H.pylori*							
*H.pylori*(−)	Case/Control	332/574	25/47	278/503	79/118	295/534	62/87
	OR(95%CI)	1(Ref)	0.92(0.55-1.52)	1(Ref)	1.21(0.88-1.66)	1(Ref)	1.29(0.90-1.83)
*H.pylori*(+)	Case/Control	469/226	36/15	407/195	98/46	425/193	80/48
	OR(95%CI)	3.61(2.93-4.45)	4.14(2.23-7.67)	3.80(3.03-4.76)	3.84(2.63-5.61)	3.97(3.18-4.96)	3.14(2.12-4.63)
		*P*_interaction_=0.592		*P*_interaction_=0.488		*P*_interaction_=0.079	
		interaction index=1.25		interaction index=0.84		interaction index=0.62	
**GC vs CON (n=724 Vs. 729)**							
*H.pylori*							
*H.pylori*(−)	Case/Control	334/484	21/44	287/429	68/99	297/456	58/72
	OR(95%CI)	1(Ref)	0.69(0.40-1.18)	1(Ref)	1.02(0.73-1.44)	1(Ref)	1.23(0.85-1.79)
*H.pylori*(+)	Case/Control	332/190	37/11	310/162	59/39	324/163	45/38
	OR(95%CI)	2.55(2.03-3.20)	4.85(2.44-9.65)	2.88(2.26-3.67)	2.25(1.46-3.46)	3.04(2.39-3.86)	1.91(1.20-3.03)
		***P*_interaction_=0.024**		*P*_interaction_=0.353		***P*_interaction_=0.031**	
		**interaction index=2.75**		interaction index=0.77		**interaction index=0.51**	

Note:

a *P* for interaction was used Logistic Regession adjusted by gender and age. CON:controls; AG: atrophic gastritis; GC:gastric cancer.

As both let-7e and miR-4795 SNPs had two-way interactions with *H. pylori* positivity, we next investigated three-way interactions of let-7e and miR-4795 SNPs with *H. pylori* infection. We found that the let-7e rs8111742 – *PGC* rs6458238 – *H. pylori* three-way combination had a positive interaction with GC risk (*P*_interactio*n* =_ 0.036, interaction index = 15.69), while the miR-4795 rs1002765 – *PGC* rs9471643 – *H. pylori* three-way combination had a positive interaction with AG risk (*P*_interactio*n* =_ 0.027, interaction index = 4.02; Table 5).

**Table 5:** The three dimensions interactions of the miRNA SNP-*PGC* SNP-*H. pylori* infection status with the risk of atrophic gastritis/gastric cancer^a^

SNP genotypes			AG vs. CON		GC vs. CON	
			*P*	OR(95%CI)	*P*	OR(95%CI)
let-7e rs8111742-*PGC* rs6458238-*H. pylori*						
GA+GG	GA+AA	(−)		1(Ref)		1(Ref)
GA+GG	GA+AA	(+)	<0.001	2.94(1.74-5.00)	0.002	2.52(1.40-4.53)
GA+GG	GG	(−)	0.772	1.05(0.72-1.55)	0.857	1.04(0.68-1.59)
GA+GG	GG	(+)	<0.001	4.20(2.83-6.22)	<0.001	2.74(1.76-4.25)
AA	GA+AA	(−)	0.128	0.20(0.03-1.59)	0.362	1.64(0.57-4.73)
AA	GA+AA	(+)	0.990	0.99(0.18-5.60)	0.465	2.11(0.29-15.52)
AA	GG	(−)	0.518	1.23(0.66-2.30)	0.059	0.42(1.17-1.03)
AA	GG	(+)	<0.001	6.82(3.00-15.49)	<0.001	8.42(3.38-21.00)
			*P*_interaction_=0.900		***P*_interaction_=0.036**	
			interaction index=0.83		**interaction index=15.69**	
let-7e rs8111742-*PGC* rs6941539-*H. pylori*						
GA+GG	CC	(−)		1(Ref)		1(Ref)
GA+GG	CC	(+)	<0.001	3.77(2.94-4.84)	<0.001	2.60(2.00-3.39)
GA+GG	CT+TT	(−)	0.074	1.31(0.97-1.77)	0.800	1.04(0.76-1.44)
GA+GG	CT+TT	(+)	<0.001	4.41(3.13-6.21)	<0.001	2.52(1.72-3.70)
AA	CC	(−)	0.928	1.03(0.56-1.90)	0.381	0.75(0.39-1.43)
AA	CC	(+)	<0.001	7.50(3.23-17.45)	<0.001	10.51(3.65-30.27)
AA	CT+TT	(−)	0.668	0.82(0.33-2.02)	0.266	0.58(0.22-1.51)
AA	CT+TT	(+)	0.215	1.88(0.70-5.06)	0.493	1.45(0.50-4.18)
			*P*_interaction_=0.265		*P*_interaction_=0.099	
			interaction index=0.37		interaction index=0.20	
miR-4795 rs1002765-*PGC* rs9471643-*H. pylori*						
GA+GG	GG+CC	(−)		1(Ref)		1(Ref)
GA+GG	GG+CC	(+)	<0.001	4.62(3.46-6.18)	<0.001	3.70(2.67-5.13)
GA+GG	GC	(−)	NA	NA	NA	NA
GA+GG	GC	(+)	<0.001	3.51(2.44-5.06)	<0.001	2.41(1.57-3.70)
AA	GG+CC	(−)	NA	NA	NA	NA
AA	GG+CC	(+)	<0.001	1.75(0.90-3.37)	0.296	1.52(0.69-3.36)
AA	GC	(−)	NA	NA	NA	NA
AA	GC	(+)	<0.001	5.14(2.24-11.82)	0.517	1.40(0.51-3.86)
			***P*_interaction_=0.019**		*P*_interaction_=0.641	
			**interaction index=4.02**		interaction index=1.40	

Note:

a *P* for interaction was used Logistic Regession adjusted by gender and age. CON: controls; AG: atrophic gastritis; GC: gastric cancer; NA, not available.

We further analyzed the ORs of the three-way interactions [let-7e rs8111742 – *PGC* rs6458238 – *H. pylori* (+) and miR-4795 rs1002765 – *PGC* rs9471643 –*H. pylori* (+)] by dividing samples into four subgroups based on the number of the interacting genotypes (Table 6). A significant dose–effect relationship was observed for the let-7e rs8111742 – *PGC* rs6458238 – *H. pylori* (+) combination: an increasing number of risk genotypes was associated with an increased risk of GC (*P*_trend_ < 0.001). A similar result was found for the miR-4795 rs1002765 – *PGC* rs9471643 – *H. pylori* (+) combination with an increased risk of AG (*P*_trend_ < 0.001).

**Table 6:** Cumulative effect of the three interacting factors of miRNA SNP-*PGC* SNP-HP infection on the gastric disease risk

No. of interacting genotypes	Total population		
	Cases/controls	*P^a^*	OR(95%CI)
let-7e rs8111742-*PGC* rs6458238-*H. pylori* on GC risk			
0	38/81		1(ref)
1	247/445	0.444	1.18(0.78-1.79)
2	204/189	<0.001	2.31(1.50-3.57)
3	28/7	<0.001	8.63(3.45-21.56)
		***P* trend<0.001**	
miR-4795 rs1002765-*PGC* rs9471643-*H. pylori* on AG risk			
0	99/188		1(ref)
1	328/467	0.043	1.34(1.10-1.77)
2	330/177	<0.001	3.57(2.63-4.84)
3	42/25	<0.001	3.51(2.00-6.17)
		***P* trend<0.001**	

Note:

a adjusted by sex and age.

### Gene-gene interaction models tested using MDR

In order to test all the above pairwises of interaction models composed of the miRNA SNPs and *PGC* SNPs, we used MDR software to verify the best model for the positive interaction which showed in Table 1. The pairwises let-7e rs8111742 – *PGC* rs6458238 and miR-4795 rs1002765-*PGC* rs9471643 combination were verified by MDR, which yielded the highest testing accuracy of 0.5288 and 0.5266, and the maximal CV consistency of 9/10 and 10/10, respectively(significant test *P* = 0.0107, and *P* for permutation test = 0.0020–0.0025 and 0.0015-0.0020, respectively). But the pairwises miR-365b rs121224-*PGC* rs6912200 and let-7e rs8111742–*PGC* rs6941539 combination were not verified by MDR, which suggested a result not stable (Supplemental Table S3).

We further verified all the three-way interaction models composed of the miRNA SNPs, *PGC* SNPs and *H. pylori*, and found they were all verified by MDR (all: significant test *P* = 0.0010, and *P* for permutation test = 0.0000–0.0005, Supplemental Table S3).

## DISCUSSION

Recently, several association studies about miRNA SNPs in gastric cancer risk have been published, including SNPs for miR-146a, miR-196a-2, miR-499, miR-149, miR-27a and miR-34b/c [16, 17]. But there is still no report about other miRNA SNPs, especially the interaction with its targeted gene SNPs. In this study, we reported for the first time about the interaction of three new miRNA SNPs and their target gene SNPs. A series of miRNA SNPs (let-7e rs8111742, miR-365b rs121224, and miR-4795 rs1002765), *PGC* target gene SNPs (rs4711690, rs6458238, rs9471643, rs3789210, rs6912200, rs6939861 and rs6941539), and *H. pylori* infection were candidate factors in first a two-way interaction and then a three-way interaction analysis. The analysis showed that two pairwise miRNA SNP – *PGC* SNP combinations contribute to AG risk. miR-365b rs121224 and *PGC* rs6912200 polymorphisms had no effect individually but an epistatic interaction for AG risk was noted when they were present together. Furthermore, two-way interactions and the addition of *H. pylori* infection as a risk factor demonstrated three-way interaction contributing to the GC and AG risks. This result suggests that the combined detection of miRNA SNPs, *PGC* SNPs, and *H. pylori* positivity is a significantly better method of detecting GC and AG risk compared with a single factor.

The effect of a single SNP on disease risk is usually reported to be weak (OR < 1.5), but combination of factors had a moderate (OR ≥ 1.5) or strong effect (OR ≥ 2) [18, 19]. Individually, all three miRNA SNPs and *PGC* SNPs had weak or no effect in the previous study: *PGC* rs4711690, rs6458238, rs3789210, and rs9471643 had weak protective effects against AG risk (OR = 0.78, 0.73, 0.78, and 0.79), and rs6939861 had a weak effect on the risk of both AG and GC (OR = 1.30 and 1.32) [11, 12]. Furthermore, the individual *PGC* rs6912200 and rs6941539 and three miRNA polymorphisms (let-7e rs8111742, miR-365b rs121224, and miR-4795 rs1002765; unpublished data) had no effect on the risk of AG or GC (i.e. did not reach statistical significance). However, in this study, these polymorphisms interacted with one another or were epistatic in the GC and AG risk. In the two-way interaction analysis, the pairwise let-7e rs8111742 – *PGC* rs6458238 combination had an OR for the interaction of 4.89 for the AG risk; this was greater than their individual effects (0.27 and 1.21, respectively). In addition, the pairwise miR-4795 rs1002765 – *PGC* rs9471643 combination had an OR for their interaction of 2.19 on AG risk; again, this was greater than their individual effects (0.80 and 0.95, respectively).

So far, several studies have shown an association between epistatic effects and cancer risk [20–22]. We also found individual SNPs at single loci that had previously shown no effect on disease risk caused epistasis when combined [23]. In this study, the strongest epistatic interaction was the pairwise miR-365b rs121224 – *PGC* rs6912200 combination. Both the rs121224 GG genotype and the rs6912200 TT genotype were associated with an increased risk of AG, but only in combination. Another two pairs of factors with epistatic effects were identified: the miR-4795 rs1002765 AA genotype was associated with a decreased risk of AG, but only in the presence of the *PGC* rs9471643 GC+CC genotype; and *PGC* rs6941539 CT+TT genotype was associated with a decreased risk of GC, but only in the presence of the let-7e rs8111742 AA genotype. These epistatic effects demonstrate that miR-365b rs121224, miR-4795 rs1002765, and the *PGC* rs6912200 and rs6941539 polymorphisms contribute to gastric carcinogenesis in a pairwise pattern. In all, this evidence suggests that the combination of miRNA SNPs and *PGC* SNPs could have a synergistic effect on gastric carcinogenesis. Concerning the possible mechanism for this SNP-SNP interaction, as miRNA could combine the 3’-untranslated region of the targeted gene, PGC protein could protect the mucosal epithelial cell [10], thus, individuals carrying the dual variation in miRNA and *PGC* gene might cause the decreasing protection function to gastric mucosa and thus increase the risk of gastric cancer. Anyway, the molecular mechanism was not clear now and further functional study would be required.

In this study, we also analyzed the interaction effect of miRNA SNPs and *H. pylori* infection on disease risk and found that two-way interactions between let-7e or miR-4795 polymorphism and *H. pylori* infection. We further analyzed the three-way interaction of miRNA SNPs, *PGC* SNPs, and *H. pylori* infection, and found that the miR-4795 rs1002765 – *PGC* rs9471643 – *H. pylori* (+) combination was positively associated with AG risk and the let-7e rs8111742 – *PGC* rs6458238 – *H. pylori* (+) combination was positively associated with GC risk. We reported that *PGC* SNPs interact with *H. pylori* infection [12], as did miRNA SNPs. This suggested that in the case of *H. pylori* infection, these specific SNPs may comprise the variant genotype associated with gastric carcinogenesis in the presence of *H. pylori*. We further analyzed the dose–effect relationship for disease risk and confirmed that the same three-way combinations were associated with GC or AG risk (*P*_trend_ < 0.001). The interaction analysis used logistic regression, and dose effects suggested that *H. pylori* infection may co-operate with miRNA SNP – *PGC* SNP to increase the disease risk. It was previously reported that PGC protein expression may be stimulated by lipopolysaccharides of the CagA (+) strain of *H. pylori* [24], supporting our evidence that any two of the three factors (i.e. miRNA SNPs, *PGC* SNPs, and *H. pylori* infection) have interaction effects; thus, our evidence suggested a biological basis for the three-way interaction. Individuals carrying these risk genotypes may be at a high risk of GC or precancerous disease; therefore, *H. pylori* should be eliminated and they should be promptly tested for the multiple risk factors to monitor gastric carcinogenesis [25].

Our study has several limitations. First, although this research already contained a relative large sample, the sample size still needs to be enlarged to confirm the interaction effect results, especially for rare genotypes. And also an independent replication is urgently encouraged for verification of our present study in the further. Second, other environmental factors and clinical pathological parameters should be included so that potential interactions between SNPs and other factors such as smoking and drinking can be analyzed.

In summary, this study found that two pairwise interacting miRNA SNP – *PGC* SNP combinations were associated with increased AG risk (let-7e rs8111742 – *PGC* rs6458238 and miR-4795 rs1002765 – *PGC* rs9471643), which were consistently identified by two different statistical approaches: multi-logistic regression and MDR analyses. In addition, the miR-365b rs121224 and *PGC* rs6912200 polymorphisms had no effect but demonstrated an epistatic effect when present together on the AG risk. Three-way interaction analysis showed that miR-4795 rs1002765 – *PGC* rs9471643 – *H. pylori* (+) had a positive interaction in AG risk and let-7e rs8111742 – *PGC* rs6458238 – *H. pylori* (+) had positive interaction in GC risk. These three-way interactions both had dose–effect correspondence. In conclusion, the miRNAs SNPs (let-7e rs8111742 and miR-4795 rs1002765) might have more superior efficiency when combined with *PGC* SNPs and/or *H. pylori* for GC or AG risk than a single SNP on its own.

## MATERIALS AND METHODS

### Patients

This study was approved by the Ethics Committee of the First Affiliated Hospital of China Medical University, Shenyang, Liaoning. The demographic and geographic characteristics of study participants are shown in Supplementary Table S1, which relates previously published data [12]. A total of 2448 individuals were recruited in this case–control study: 862 AG patients and 862 healthy individuals matched by age and sex (control group); and 724 GC patients. For GC risk analysis, 729 individuals in the control group matched by age and sex were selected to compare with the GC group. Healthy control and AG tissue samples were selected from a GC screening program that took place in the Zhuanghe area of Liaoning Province between 1997 and 2008. GC patients were recruited individuals who underwent surgery at the First Affiliated Hospital of China Medical University. Informed consent was obtained from all participants and medical information (included age, sex) was collected by questionnaire survey or interview. Peripheral venous blood samples were taken from all participants, and all underwent gastroscopic examination and biopsy collection; disease was confirmed by a histopathological diagnosis by two independent pathologists. The diagnosis for the control group was normal or slight superficial gastritis and AG was diagnosed used the Sydney classification [26, 27]. The inclusion criterion for the AG group was histologically confirmed medium or severe AG [28].

### SNP genotyping

Genomic DNA was extracted from venous blood as previously described [29]. The DNA concentration was adjusted to 20 ng/μl. SNP genotyping was performed using Matrix assisted laser desorption/ionization time of flight mass spectrometry, as previously described [12]. Ten percent of all samples were re-analyzed to verify the result: the consistency rate was higher than 99%.

### Detection of the serum H. pylori IgG titer

We tested the serum *H. pylori* immunoglobulin G (IgG) antibody titer by enzyme-linked immunosorbent assay (*Helicobacter pylori* IgG kit; Biohit, Helsinki, Finland) according to a previously described method [30, 31]. Patients with a serum titer > 34 IU were diagnosed as *H. pylori* positive.

### Statistical analysis

This study defined the heterozygote plus rare homozygote compared with the wild-type genotype as the dominant model, the mutant genotype compared with the wild-type genotype plus the heterozygote as the recessive model, and the heterozygote compared with the wild-type plus the mutant genotype as the complete overdominant model [15]. The distribution of demographic characteristics and the genotype in case and control groups was tested using χ^2^ test. Multivariate logistic regression analysis adjusted by age, sex, and *H. pylori* status was used to assess the OR and 95% confidence interval for the GC and AG risks. The log likelihood ratio test was used for the interaction analysis between miRNA SNP, *PGC* SNP, and *H. pylori* status. The Cochrane–Armitage test for linear trends was used to judge the dose–effect relationship between GC/AG risk and an increasing number of risk genotypes. All the risk factors including miRNA SNPs, *PGC* SNPs and *H. pylori* identified in the best models of gene–gene interactions were calculated using MDR software (version 3.0.2) and MDR permutation testing software (version 1.0 beta 2) [32] in order to confirm the multivariate logistic regression results. All statistical tests were performed using PASW Statistics for Windows, Version 18.0 (SPSS Inc., Chicago, IL, USA) and Stata version 11.0 (StataCorp, College Station, TX, USA). All tests were two sided and statistical significance was set at *P* < 0.05.

## SUPPLEMENTARY TABLES



## Abbreviations

CON: Control
AG: Atrophic Gastritis
GC: Gastric Cancer
SNP: single nucleotide polymorphism
H. pylori: Helicobacter pylori
