# Supplementary material for: SNP-SNP interactions of three new pri-miRNAs with the target gene PGC and multidimensional analysis of H. pylori in the gastric cancer/atrophic gastritis risk in a Chinese population
Source: Oncotarget. 2016 Mar 14;7(17):23700–14. doi: 10.18632/oncotarget.8057 (PMC5029657; doi:10.18632/oncotarget.8057)
Supplement: Supplementary file 1 [file oncotarget-07-23700-s001.pdf]

SNP–SNP interactions of three new pri-miRNAs with the target gene *PGC* and multidimensional analysis of *H. pylori* in the gastric cancer/atrophic gastritis risk in a Chinese population

SUPPLEMENTARY TABLES

Supplementary Table S1: The baseline of the subjects

Variables	AG vs. CON		GC vs. CON	
	**CON(%)**	**AG(%)**	**CON(%)**	**GC(%)**
	**n=862**	**n=862**	**n=729**	**n=724**
Gender	*P*=0.846		*P*=0.564	
Male	483(56.0)	487(56.5)	483(66.3)	490(67.7)
Female	379(44.0)	375(43.5)	246(33.7)	234(32.3)
Age	*P*=0.343		*P*=0.562	
Mean±SD	54.9±9.2	55.4±9.5	56.1±9.2	56.4±9.8
Median	54	56	56	57
Range	17-85	16-79	17-85	21-81
H. pylori	*P*<0.001		*P*<0.001	
Positive	241(28.0)	505(58.6)	201(27.6)	369(51.0)
Negative	621(72.0)	41.4(36.5)	528(72.4)	355(49.0)

Supplementary Table S2: The interaction of miRNA SNP-miRNA SNP in the risk of gastric cancer/atrophic gastritis[a]

		let7e rs8111742		miR-365b rs121224	
		**GA+GG**	**AA**	**CG+CC**	**GG**
**AG vs CON (n=862 Vs. 862)**					
miR-365b rs121224					
CG+CC	Case/Control	634/649	51/49	/	/
	OR(95%CI)	1(Ref)	1.06(0.71-1.59)	/	/
GG	Case/Control	167/151	10/13	/	/
	OR(95%CI)	1.13(0.88-1.44)	0.78(0.34-1.80)	/	/
		$P_{interaction}$=0.244		/	
		interaction index=0.55			
miR4795 rs1002765					
GA+GG	Case/Control	668/671	52/56	569/595	151/132
	OR(95%CI)	1(Ref)	0.93(0.63-1.38)	1(Ref)	1.19(0.92-1.55)
AA	Case/Control	133/129	9/6	116/103	26/32
	OR(95%CI)	1.05(0.80-1.37)	1.50(0.53-4.24)	1.20(0.90-1.60)	0.85(0.50-1.44)
		$P_{interaction}$=0.636		$P_{interaction}$=0.196	
		interaction index=1.33		interaction index=0.64	
**GC vs CON (n=724 Vs. 729)**					
miR-365b rs121224					
CG+CC	Case/Control	551/546	46/45	/	/
	OR(95%CI)	1(Ref)	1.01(0.66-1.54)	/	/
GG	Case/Control	115/128	12/10	/	/
	OR(95%CI)	0.88(0.67-1.17)	1.18(0.51-2.76)	/	/
		$P_{interaction}$=0.368		/	
		interaction index=1.59			
miR4795 rs1002765					
GA+GG	Case/Control	569/568	52/51	512/511	109/108
	OR(95%CI)	1(Ref)	1.01(0.68-1.52)	1(Ref)	1.00(0.75-1.35)
AA	Case/Control	97/106	6/4	85/80	18/30
	OR(95%CI)	0.93(0.69-1.25)	1.49(0.42-5.32)	1.08(0.78-1.51)	0.60(0.33-1.08)
		$P_{interaction}$=0.368		$P_{interaction}$=0.155	
		interaction index=1.44		interaction index=0.58	

Note:[a], *P* for interaction was used Logistic Regession adjusted by gender, age and *H. pylori* infection status. CON:controls; AG: atrophic gastritis; GC:gastric cancer.

Supplementary Table S3: MiRNA-*PGC* gene interaction models for polymorphisms and for *H. pylori* in gastric cancer and/or atrophic gastritis risk by MDR analysis

Model	Training Bal. Acc.	Testing Bal. Acc.	Sign Test (p)	CV Consistency	*P* for permutation test
For AG risk					
Two-way interaction					
let-7e rs8111742-PGC rs6458238	0.5344	0.5288	**9(0.0107)**	9/10	**0.0020-0.0025**
miR-4795 rs1002765-PGC rs9471643	0.5397	0.5312	**9(0.0107)**	10/10	**0.0015-0.0020**
miR-365b rs121224-PGC rs6912200	0.5127	0.4780	3(0.9453)	7/10	0.9660-0.9665
let-7e rs8111742-PGC rs6941539	0.5259	0.5052	5(0.6230)	8/10	0.5390
Three-way interaction					
let-7e rs8111742-PGC rs6458238-*H. pylori*	0.6707	0.6678	**10(0.0010)**	5/10	**0.0000-0.0005**
let-7e rs8111742-PGC rs6941539-*H. pylori*	0.6547	0.6500	**10(0.0010)**	10/10	**0.0000-0.0005**
miR-4795 rs1002765-PGC rs9471643-*H. pylori*	0.6720	0.6630	**10(0.0010)**	5/10	**0.0000-0.0005**
For GC risk					
let-7e rs8111742-PGC rs6458238-*H. pylori*	0.6883	0.6854	**10(0.0010)**	10/10	**0.0000-0.0005**

Note: When the *P* value for the Sign Test <0.05 and *P* for permutation test <0.05 meant a statistical difference. AG, atrophic gastritis; GC, gastric cancer.
